# Historical determinants of neurosurgical inequities in Africa and the African diaspora: A review and analysis of coloniality

**DOI:** 10.1371/journal.pgph.0001550

**Published:** 2023-02-06

**Authors:** Ernest J. Barthélemy, Sylviane A. Diouf, Ana Cristina Veiga Silva, Nancy Abu-Bonsrah, Isabella Assunção Santos de Souza, Ulrick Sidney Kanmounye, Phabinly Gabriel, Kwadwo Sarpong, Edjah K. Nduom, Jean Wilguens Lartigue, Ignatius Esene, Claire Karekezi

**Affiliations:** 1 Global Neurosurgery Laboratory, Division of Neurosurgery, Department of Surgery, SUNY Downstate Health Sciences University, Brooklyn, New York, United States of America; 2 Society of Haitian Neuroscientists, Inc., New York, New York, United States of America; 3 Center for the Study of Slavery & Justice, Brown University, Providence, Rhode Island, United States of America; 4 Neurosurgery Service, Clinical Hospital, Federal University of Pernambuco, Recife, Brazil; 5 Department of Neurosurgery, Johns Hopkins University School of Medicine, Baltimore, Maryland, United States of America; 6 Research Department, Association of Future African Neurosurgeons, Yaoundé, Cameroon; 7 Bahia School of Medicine and Public Health, Salvador, Brazil; 8 Department of Neurosurgery, University of Alabama at Birmingham, Birmingham, Alabama, United States of America; 9 Department of Neurosurgery, Vanderbilt University Medical Center, Nashville, Tennessee, United States of America; 10 Department of Neurosurgery, Emory University School of Medicine, Atlanta, Georgia, United States of America; 11 Department of Surgery, Mirebalais University Hospital, Zanmi Lasante, Mirebalais, Haiti; 12 Department of Neurosurgery, Division of Neurosurgery, Faculty of Health Sciences, University of Bamenda, Bamenda, Cameroon; 13 Neurosurgery Unit, Department of Surgery, Rwanda Military Hospital, Kigali, Rwanda; University of Cape Town, SOUTH AFRICA

## Abstract

The movement to decolonize global health challenges clinicians and researchers of sub-disciplines, like global neurosurgery, to redefine their field. As an era of racial reckoning recentres the colonial roots of modern health disparities, reviewing the historical determinants of these disparities can constructively inform decolonization. This article presents a review and analysis of the historical determinants of neurosurgical inequities as understood by a group of scholars who share Sub-Saharan African descent. Vignettes profiling the colonial histories of Cape Verde, Rwanda, Cameroon, Ghana, Brazil, and Haiti illustrate the role of the colonial legacy in the currently unmet need for neurosurgical care in each of these nations. Following this review, a bibliographic lexical analysis of relevant terms then introduces a discussion of converging historical themes, and practical suggestions for transforming global neurosurgery through the decolonial humanism promulgated by anti-racist practices and the dialogic frameworks of conscientization.

## Introduction

Global Neurosurgery (GN) is a multidisciplinary subspecialty of global surgery that sits at the nexus of neurosurgery and public health, and aims to remedy the world’s unmet need for neurosurgical care. Grounded in health equity, GN aims to overcome the impact of sociohistorical phenomena that have generated disparities between people who can access essential neurosurgical care, and those who cannot [1, 2]. The public health community is currently experiencing an intensifying call to “decolonize” global health (GH) as the COVID-19 pandemic has coincided with a contemporaneous public health crisis of systemic racism [3–8]. This moment of global reckoning has reintroduced into public health and increasingly, popular culture, a vocabulary of social justice formerly limited to the social sciences, e.g., structural racism, structural violence, anti-racism, etc [9–12]. The introduction of such terms into the modern GN vernacular will likely shape the subspecialty’s evolution. Understanding the historical determinants of neurosurgical inequities, and the relationships of this emerging language to these determinants, can empower GN practitioners to transform GH through comprehensive and enduring decolonization.

This article aims to educate stakeholders in the GN ecosystem about the colonial legacy through the analytical lens of neurosurgeons and scholars of Sub-Saharan African descent. Focusing primarily on extractive colonialism, the transatlantic slave trade, and their putative role in establishing the disparities driving the unmet need for neurosurgical care throughout Sub-Saharan Africa and the African Diaspora, we provide a historical review and analysis that may empower GN practitioners to recognize, attenuate and dismantle neo-colonial practices in modern GN. Brief vignettes highlight the impact of colonialism and slavery-based economies on healthcare and neurosurgery in Cape Verde, Brazil, Ghana, Haiti, Cameroon, and Rwanda. We then define and analyse terms of interest in the decolonization movement and conclude with reflections and recommendations on how practitioners may begin decolonizing GN.

As noted above, we have deliberately focused this narrative specifically on the history of colonialism and repercussions of the colonial legacy as determinants of current neurosurgical inequities in several countries of Africa and two Latin American countries of the African Diaspora. As a result of this prism of analysis, discussion of several other important determinants of these inequities, and potential solutions thereof, have been deferred to future study. The authors acknowledge, however, that these determinants include the primordial roles of governance, sovereignty, and the agency of local African and African Diaspora leaders regarding accountability for existing disparities in their nations, and ultimate responsibility for generating solutions that serve the nations they lead.

## Methods

### Ethics committee approval

This study entailed no human subjects research and was therefore exempt from ethics committee approval.

### Comparative workforce density assessment

Utilizing neurosurgeon workforce density per 100,000-population (NWD) as a proxy for neurosurgical capacity, we assessed the impact of former colonial relationships on current global neurosurgical inequities by comparing the NWD of the low- and middle-income countries (LMICs) in our review to the NWD of their former metropolises, which have invariably become high-income countries (HICs). The Global Neurosurgical Workforce Map of the World Federation of Neurosurgical Societies was utilized for this assessment, and supplemented with data from the literature providing more recent NWD data where available [13–29].

### Search strategy and selection criteria

We performed a historical, narrative review of the literature exploring interrelationships between colonialism and modern disparities in GN. Research teams were organized according to the authors’ ethnicities, and relevant experiences in their home countries of interest, including Cape Verde, Brazil, Ghana, Haiti, Cameroon and Rwanda. Databases searched included PubMed, JSTOR, SciELO, LILACS and internet searches via Google and Google Scholar. Search terms included the above-named countries and relevant sociohistorical terms such as *slavery*, *racism*, *colonialism*, *middle passage*, and *transatlantic slave trade*. We explored the relevance of these concepts to GN by using the Boolean operator “AND” to link them to the search terms *medicine*, *surgery*, *neurosurgery*, *GH* or *public health*. Search results that were relevant to the sociohistorical themes of colonial history, global neurosurgery and public health were included, and irrelevant results excluded. Results were then curated to generate vignettes that facilitate a comparative study of the colonial history of GN. Vignettes were critically reviewed and edited by a research scholar with specific expertise in the colonial history of Sub-Saharan Africa and the African Diaspora (S.A.D.).

### Bibliometric analysis

We performed a bibliometric analysis and literature search aimed at defining a decolonial lexicon from the fields of anthropology and social medicine. Five key terms were selected and defined for this analysis by author consensus based on major themes from recent and relevant literature [9, 11, 30–38]. The contemporary biomedical relevance of these terms was explored by searching each term as an exact phrase in PubMed, and graphically representing the annual number of search results. The terms are subsequently integrated into a discussion and recommendations for a decolonial approach to modern GN research and practice.

## Results

Current GN disparities are rooted in the legacy of colonialism and the transatlantic slave trade. Table 1 illustrates these disparities by comparing the NWD and related data from Cape Verde, Brazil, Ghana, Haiti, Cameroon and Rwanda, to their respective former metropolises. This assessment contextualizes the ensuing vignettes by quantifying current differences in neurosurgical capacity as one impact of extractive colonialism, where extraction included human resource exploitation in the form of slave-trade and slavery-based economies in Africa and the Americas [i.e., they were societies *with* slaves, or from which people were taken and *en*slaved, but were not *slave* societies], and not only material resource extraction.

**Table 1:** Comparison of current neurosurgical workforce.

Former Colonial Power/Current HIC					Former Colony/Current LMIC				
Nation	Pop (x1M)	#NS	NWD	YTS	Nation	Pop (x1M)	#NS	NWD	YTS
France	66·496	443	0·666	1938 [14]	Haiti	11·198 [15]	3	0·028	2016 [16]
					Cameroon	24 [17]	26 [17]	0·108	2010 [17]
United Kingdom	67·081 [18]	389 [19]	0·580	1933 [20]					
					Ghana	30·1 [17]	24 [17]	0·080	1989 [21]
Belgium	11·231	158	1·406	1948 [22]	Rwanda	12·956 [23]	6	0·046	2012 [24]
Germany	80·983	1285	1·586	1934 [25]					
Portugal	10·401	168	1·615	1955 [26]	Cape Verde	0·514	2	0·389	N/A
					Brazil	212·559 [27]	3682 [28]	1·73	1970 [29]

Comparison of current neurosurgical workforce: Former colonial power vs. former colony. Former colonial metropolises which are currently High-Income Countries (HIC) are matched to their former colonies which are currently low- and middle-income countries (LMIC). Where an HIC historically colonized more than one nation, its row intersects with both former colonies, e.g., France and Portugal. Where LMIC has been colonized by more than one HIC, it has been positioned so that its row appears at the intersection of its corresponding former metropolises, e.g., Cameroon and Rwanda. Legend: Pop (x1M) = Population in millions. #NS = quantity of neurosurgeons. NWD = Neurosurgeon Workforce Density per 100,000-population. YTS = Year neurosurgery training began. Data is from the 2016 WFNS Neurosurgery Workforce map except where indicated.

Neurosurgical inequities in LMICs descend from economic destabilization with two major socio-historical aetiologies: (1) the economic logic of the colonial legacy as described below, and (2) postcolonial development policies that crippled LMIC economies through neo-colonial practices such as structural adjustment programs [39]. Structural adjustment programs are conditional lending measures implemented in LMICs by the Bretton Woods (BW) financial institutions of the United Nations, i.e., the World Bank and the International Monetary Fund, intending to promote economic health and sustainable development in the impoverished nations of Africa and Latin America [40, 41]. These structural adjustment programs are instead widely believed to have resulted in systematic decentralization, defunding and privatization of the public sector in LMICs, with disastrous health-related, education-related, and sociocultural consequences for citizens in these nations [42]. While comprehensive analysis of the impact of BW institutions on neurosurgical care in LMICs is beyond the scope of this article, the ensuing vignettes will lay a foundation for understanding the colonial roots of modern GH policy, much of which has been shaped by the BW Institutions.

### Vignettes

#### Cape Verde

Cape Verde is a 10-island archipelago of Sub-Saharan Africa located about 450 km off the Senegalese coast with approximately 500,000 inhabitants [43]. Portuguese colonizers arrived in 1460, converting the archipelago into, “…an open-air hypermarket” for slave commerce [44]. Indeed, slave trade and geostrategy constituted the principal objectives of colonization [45–47]. The socioeconomic and cultural repercussions of this colonial legacy were profound, lasting over 400 years. This period indissolubly linked the archipelago’s destiny to the African mainland, Europe and the Americas through the Transatlantic slave trade; it functioned as a living, mercantile laboratory for experimentation with animals, plants and human beings [48].

Cape Verde has been dubbed a “success story” in African development. Strong governance, prudent resource management, and economic growth stimulated by the Cape Verdean Diaspora have facilitated health system achievements e.g. improvements in life expectancy, maternal and child health, and managing infectious diseases [43]. Nonetheless, structural vulnerabilities such as lack of essential medications, infrastructure, equipment, and specialized physicians such as neurosurgeons, obligate Cape Verdeans to resort to dependence upon humanitarian aid, interisland medical transfers or evacuations abroad for neurosurgery as well as other subspecialty care, through a complex, bureaucratic process that often burdens patients socioeconomically [45–47]. In particular transfers for neurosurgical care have accounted for 6–10% of evacuations over the past three years (Table 2).

**Table 2:** Cape Verdean transfers.

Evacuation Type	Year: 2020	Year: 2019	Year: 2018
**Inter-island**	2644	2572	3223
**Abroad**	229	319	410
**Neurosurgery**	20/229	25/319	28/410
**Returns**	172	256	252
**Deaths**	27	23	22

Cape Verdean transfers and medical evacuations by year, inter-island and abroad, per the National Social Security Institute reports [49].

Cape Verde’s current NWD of 0·39 stems from two Cuban neurosurgeons, the first of whom joined the National Health System in 2014. The contributions of both neurosurgeons are vital, yet inadequate to meet the country’s neurosurgical disease burden, especially as eight of the nine inhabited islands still lack even one neurosurgeon.

#### Brazil

Portugal’s principal wealth-generating colonial enterprise was a systematic exploitation of 4·9 million Africans transported to Brazil from the time of Portuguese Maritime Expansion through abolition of slavery in 1888 [50, 51]. Preceding this, several movements tended towards emancipation, e.g. the Malê Revolt of Salvador in 1835 led by African Muslim freedom fighters. During this period, African descendants represented 78% of the population; 40% of them were slaves [52]. Currently, Salvador is the Brazilian city most densely populated by African descendants who are racially characterized as “black,” and experience profound social inequity [53]. In 2018, the average income of black people was 32% of their white counterparts’ income, revealing the socioeconomic dominance of a white Brazilian minority [53]. Structural racism resulting from Brazil’s colonial history was exemplified by the creation of “Land Laws,” which were legislative attempts by dominant landowners to prohibit black ownership of property [54].

Brazil’s colonial legacy of racism persists in medicine: graduating medical students in 2019 were 67·1% white, 24·3% brown, 3·4% black, and 5·2% of undeclared or other race/ethnicity [28]. By comparison, racial demographics for the nation included: 46·7% brown, 44·2% white and 8·2% black [55]. “Whites” were therefore overrepresented in medicine by 52%, while “browns” and “blacks” were *under*represented by 48% and 59%, respectively. Similar disparities persist in neurosurgery through inequitable countrywide distribution of Brazil’s 3,682 neurosurgeons: the predominantly white state of São Paulo has an NWD of 3·71, which is over double the NWD of the world’s wealthiest economies [13]. By contrast, the predominantly black state of Bahia has an NWD of 0·74, translating into less than 20% of São Paulo’s NWD [28]. The black and brown populations predominate in the North and Northeast of the country, which concentrate respectively 5·2% and 15·4% of Brazilian neurosurgeons, and have the lowest NWD [28]. Thus, racial/ethnic inequities in Brazilian neurosurgery are evident in both the disproportionate underrepresentation of black and brown African descendants in Brazil’s neurosurgery workforce, as well as the related geographic disparities of access to neurosurgical care that adversely affect regions more densely populated by Brazilians of predominantly African descent. While the causes of these neurosurgical inequities are likely multi-factorial, the legacy of Brazil’s history of slavery and colonialism informs structural racism against Brazil’s African descendants that contributes to these inequities.

#### Ghana

Ghana is a West African country with about 31 million inhabitants. European colonizers dubbed Ghana the Gold Coast upon their arrival in the 1400s [56]. The Portuguese were the first to stake their claim on the colony, followed in quick succession by Dutch, Danish, French, and British colonizers who exploited the country’s human (via slavery) and natural resources (gold, bauxite, timber) [57]. Ghana became the first independent post-colonial African nation in in March 1957 under Dr Kwame Nkrumah’s leadership. However, the post-independence renaissance was short-lived with Nkrumah’s overthrow in 1966, followed by a series of military-style leaders until1992 when Jerry Rawlings, the last of the military dictators, instituted democratic elections [56].

Throughout Ghana’s development, healthcare delivery has been fraught with challenges including inadequate human resources and infrastructure [58]. With increasing healthcare demands in the post-independence era, and neo-colonial pressure from the BW institutions to meet the conditions of government financing assistance, Ghana implemented a user fee-driven health financing strategy called “cash-and-carry” [59, 60]. Despite data suggesting that the system advanced development, cash-and-carry increased financial burden and worsened health outcomes for vulnerable populations [59, 60]. In some instances, all anticipated hospital fees required prepayment; otherwise, patients were sequestered in hospitals until fees were paid. In the early 2000s, the National Health Insurance Scheme was established to mitigate these costs, though patients continue making significant out-of-pocket payments for care [58, 61, 62]. Additionally, care delivery is complicated by poor infrastructural support and inadequacy of trained personnel, diagnostic tests, imaging and surveillance [63]. Patients generally seek care at hospitals, though many consult traditional healers either as an adjunct or alternative to Western medicine [64, 65].

In neurosurgery, health system limitations are further compounded by resultant frustrations for the country’s 24 neurosurgeons and the patients they serve [17]. Ghana’s low NWD is concentrated in a few urban hospitals, and attributable to several workforce development barriers, e.g. the lengthy structure of training which is influenced by the British educational system [17]. Ghana’s neurosurgery training pathways include the programs of the West African College of Surgeons or the Ghana College of Physicians and Surgeons [17]. Lack of neurosurgical capacity has led to delayed or inappropriate care for patients and limited access to diagnostics and post-acute rehabilitation. In this manner, the colonial legacy has continued to drive neurosurgical inequity for most Ghanaians.

#### Haiti

In 1492, Columbus’s landing on Hispaniola ignited a 200-year period marked by the genocide of Taino Arawaks by Spanish colonizers, the systematic importation and enslavement of Africans, and the arrival of competing European forces like France [66–68]. After gaining the western third of the island in 1697, France continued to import African slaves in the renamed colony of Saint Domingue for its wealth-generating sugar plantation business [67]. Slavery ended a century later when African slaves and their descendants revolted against France, creating the Republic of Haiti on January 1, 1804 [69].

Haiti’s revolution resulted in westward territorial expansion of the U.S. through the 1803 Louisiana Purchase. Nonetheless, France and the U.S. refused to recognize Haiti’s sovereignty until 1825 and 1862, respectively [70]. Postcolonial relationships between Haiti and these superpowers systematically undermined Haiti’s viability. France’s recognition required Haiti to pay a reparations indemnity for loss of slavery-based sugar business; currently valued at $21 billion USD, Haiti completed payment in 1947 [71, 72]. U.S.-Haiti relations were punctuated by similar neo-colonialism, including periods of US military occupation and support for dictatorial regimes [73, 74].

Haiti’s resulting struggles include an impoverished neurosurgical landscape from inadequate training opportunities and healthcare investment [75, 76]. Neurotrauma, stroke and hydrocephalus are major causes of morbimortality harkening the need for access to neurosurgical care [77]. Only three neurosurgeons currently serve Haiti’s 11 million inhabitants, and sparse resources like neuroimaging hinder care while obscuring the real epidemiology of neurosurgical disease [76, 78, 79]. Despite recent humanitarian projects led by foreigners, the colonial impact on Haitian neurosurgery has persisted, now characterized by NGO-dependency. High-income countries like the US, Canada and France still finance projects that prioritize their interests over the national interests of Haiti’s public sector, thus perpetuating neo-colonial barriers to sustainable development. Haiti’s economically fragile and vulnerable public sector consequently fails to deliver comprehensive neurosurgical care to all Haitians who need that care, even as some foreign NGO projects manage to intermittently deliver such care to some, more fortunate Haitians. While comprehensive understanding of the drivers of neurosurgical inequities in Haiti will require further analysis of the political economy of Haitian neurosurgery, that analysis must be grounded in a study of the contemporary repercussions of Haiti’s colonial history. Global neurosurgery initiatives that seek to address Haiti’s neurosurgical inequities will therefore require decolonial partnerships that empower local capacities for neurosurgical care [76, 80].

#### Cameroon

Cameroon’s health system, including the country’s ability to provide neurosurgical care, is intrinsically linked to Cameroonian colonial history. Germany annexed Cameroon’s littoral regions in 1884 using evangelism, trade, and military domination. The colonizers signed exploitative treaties with traditional rulers and renamed the region Kamerun. Following the First World War, Kamerun came under British and French rule, and was geographically partitioned to accommodate the new colonial powers. Western Kamerun was annexed to British Nigeria and became English-speaking Cameroon, while eastern Kamerun became Francophone Cameroun under France. The differing German, English, and French brands of colonization had varying impacts on the health system. British rule utilized pre-existing power structures, whereas France imposed its culture and values through forced assimilation [81]. The two resulting Cameroons developed dissimilar levels of autonomy, infrastructure, and sustainability between 1918 and 1960.

In French Cameroun, local healthcare workers were marginalized and traditional medicine was discouraged, which had serious implications for neuroscience because neurological disorders are often believed to be metaphysical rather than biological [82]. This alienation of local and traditional healers from colonial health structures adversely impacted health-seeking behaviours and contributed to care-seeking delays for Cameroonian patients with neurosurgical disorders since patients requiring neurosurgical care often sought the care of such healers first [83]. Christianity was central to healthcare throughout both sides of Cameroon, as pastoral outposts were the administrative bases for colonial interventions and religious facilities long served as the sole primary care centres in most communities. Moreover, these outposts were laboratories for human experimentation up until independence and set the stage for abuses and significant health disparities [84]. Christian health facilities still play a critical role in delivering nearly one-third of care, which likely explains the health disparities of the three majority Muslim Northern regions despite that they comprise nearly 40% of the population [85].

When the two Cameroons gained independence and unified, the larger francophone Cameroun became dominant, extending the French administrative and healthcare blueprint over its anglophone counterpart. Power and decision-making were concentrated in the capital, Yaoundé, where a single medical school trained physicians mostly in French for decades following independence. Despite offering surgical training, lack of neurosurgery training long obligated aspiring neurosurgeons to emigrate. Moreover, socioeconomic vulnerabilities of referral hospitals outside of the Centre and Littoral regions, resulting in part from Cameroon’s colonial legacy, have limited infrastructure and inadequate funding and authority to increase access to neurosurgery. As a result, patients from eight of the ten regions of Cameroon, must travel long distances to get care from one of the country’s 26 neurosurgeons in Yaoundé or Douala [17, 83]. Understanding the legacy and contemporary repercussions of Cameroon’s shared French, British and German colonial history is therefore vital to global neurosurgery initiatives that aspire to advance equitable neurosurgical care for all people of Cameroon.

#### Rwanda

Rwanda, a landlocked East African country with approximately 13 million people, was a German colony from 1897 to 1919. Following World War II, it was placed under Belgian mandate [86–88]. Belgian health policy was mainly organized in an authoritarian way [89]. Recurring famines weakened the region. Maternal and child mortality reached alarming levels. Many endemic diseases affected the population and deadly epidemics followed. Living conditions were harsh with rudimentary hygiene, in a context of almost generalized poverty. In 1985, Rwanda adopted a health development strategy based on decentralization and district-level care [90]. Decentralization began with the development of provincial-level health offices for health system management. The country’s progression was completely disrupted by the 1994 Genocide that took over 800,000 lives. The health sector was also compromised by loss of health professionals and destruction of much of the health system and its infrastructure.

Rwanda has made remarkable progress towards recovery characterized by increased stability, rebuilt national security, and multisectoral reinvigoration, including healthcare. In February 1995, the government issued a new policy to guide health system reconstruction. Rwanda surpassed many of its pre-1994 health targets, including reduction in HIV/AIDS prevalence, under-five mortality and road traffic accidents. Health insurance became mandatory for all individuals in 2008; in 2010 over 90% of the population was covered [91]. In 2012, only about 4% were uninsured. Nevertheless, Rwanda continues to face a high disease burden, with mortality stemming primarily from complications of HIV/AIDS, severe malaria, pulmonary infections, and trauma. Traditional medicine remains also widely used. Sick people are as likely to consult traditional practitioners as their modern healthcare providers, depending on the nature of the problem [92].

The University of Rwanda (formerly National University of Rwanda) restarted medical school in 1996 and its first surgery training program in 2005. Before then, such specialists were trained abroad, mainly in South Africa, West Africa, East Africa and Europe [24]. Until recently, major neurosurgical procedures could not be performed and patients required referral abroad. Numerous salvageable patients died due to non-operated neurotrauma, while undiagnosed brain and spinal conditions remained common [24]. Previous neurosurgical care was provided by foreign surgeons. In 2007, the country had only one CT scanner, no MRI, and only one Rwandan neurosurgeon. In 2010, a 1.5 Tesla MRI was serving the entire nation. In 2012 a local postgraduate training program in neurosurgery was initiated [24]. By December 2021, the country counted six neurosurgeons and some trainees.

### Bibliometric analysis

**Fig 1:**
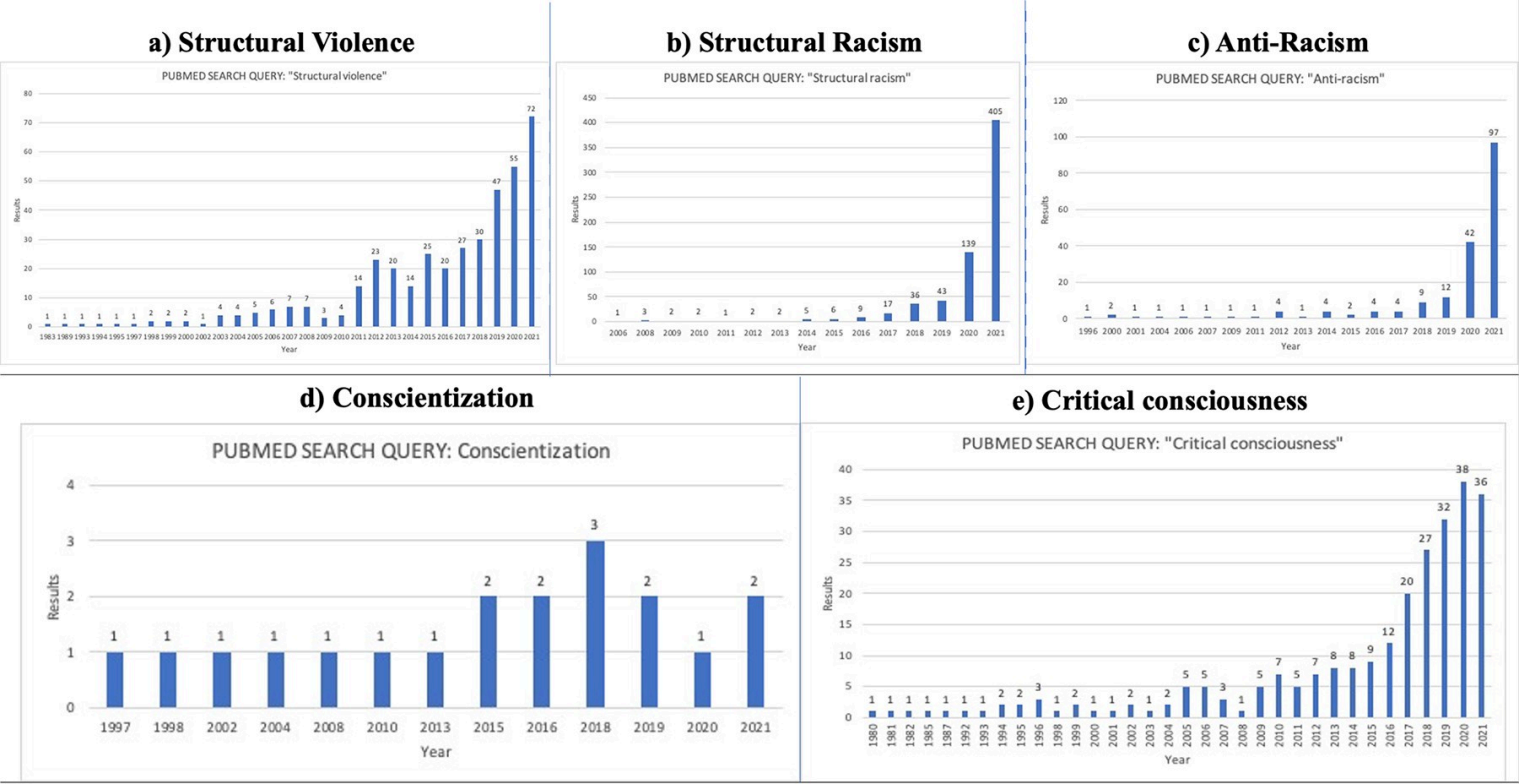
a-e. Results of bibliometric analysis on terms of relevance in the decolonisation movement. See text for methods.

The narratives above reveal the ubiquity of the colonial legacy in several countries of Africa and the African Diaspora, and the impact of this legacy on these countries’ modern health system inequities, including those of GN and other subspecialties of global surgery. Results of our accompanying lexical and bibliometric analysis are outlined below and presented in Table 3 and Fig 1A–1E.

**Table 3:** A decolonial lexicon for global neurosurgery.

Term	Definition/Description
**Structural Violence **	Structural violence refers to harm that comes to people who, by virtue of social structures, governments, economies, religions, laws or policies, face significant barriers to having their basic needs met. Examples of these barriers include institutional or structural racism, elitism, ethnocentrism or nationalism [93]. It is therefore distinguished from other forms of violence by (1) the relative invisibility of its source and (2) the absence of one or more identifiable aggressor(s).
**Structural Racism **	Structural racism refers to legislation, policies and societal norms that are based on facilitating economic advantage and prosperity for members of a favoured race, such as people of “fair” or “lighter” skin colour who appear to descend from Western Europe, while codifying barriers to prosperity and access for people of darker skin colour who appear to descend from Sub-Saharan Africa [11, 94].
**Anti-Racism**	As the term implies, anti-racism refers to approaches that expose discriminatory perspectives, policies, practices and behaviours that are based on racial differences. Anti-racist approaches advance health equity by prioritizing solutions that favour groups of people who suffer most because of their racial background or identity [34].
**Conscientization** **and** **Critical Consciousness**	The related terms “conscientization” and “critical consciousness” are concepts from the field of adult education advanced by the late Brazilian philosopher and educator, Paolo Freire, in Pedagogy of the Oppressed, and related work on transformative education. Critical consciousness refers to a reflective awareness of the differential power dynamics and privileges that underlie societal inequities [95] The development of this form of awareness is a process that Freire calls, “conscientization,” and is distinguished as an approach to education that liberates participants in the educational process rather than perpetuating the “oppressor/oppressed” dichotomy in traditional forms of education [30].

A Decolonial Lexicon for Global Neurosurgery. Terms were selected by the authors based on their recurrence in relevant literature as emerging themes in the decolonization movement [9, 11, 30–38].

#### Structural violence

Harm resulting from neurosurgical inequities is a form of, “structural violence” [10]. The term more than doubled its frequency of occurrence in the biomedical literature of 2021 (72 results) versus three years prior (Fig 1A). Structural violence in GN can occur when social determinants of health such as race, occupation, or domiciliary status directly impact *whether* people suffer from neurosurgical disorders, the *severity* of disease at the time of diagnosis, (3) *availability* of therapy and (4) patient outcomes.

#### Structural racism

Since race is not biological, but rather a socially constructed classification based on natural variations in human appearance, the rarity of explicit race-based discrimination poses a challenge to decolonization because the latter seeks to dismantle phenomena that are not universally acknowledged to exist [37]. Nonetheless, the vignettes above illustrate how coloniality has structurally shaped current racial disparities that impact all areas of global health, including GN. Understanding the institutionalization of the racist premises of colonialism in modern health systems is therefore vital to decolonization, and may explain the growing use of the term, “structural racism” in the biomedical literature (Fig 1B).

#### Anti-racism

The term, “Anti-racism,” is defined in Table 3, and was observed to be utilized with a nearly 10-fold increase in frequency of occurrence in the biomedical literature of 2021 compared to only two years prior (Fig 1C). Anti-racism may include promoting leadership in GN by practitioners whose demographics match those of the community served. Such approaches can radically transform structural racism by mitigating mistrust from marginalized communities and strengthening the cultural effectiveness of GH workers and the teams they lead [34].

#### Critical consciousness/Conscientization

“Critical consciousness” and “conscientization,” used interchangeably here, are approximate translations of the Portuguese term, *conscientização*, promulgated by the Brazilian education scholar, Paolo Freire [33, 35]. While “critical consciousness” is the term of increasing frequency, “conscientization” will be used below (Fig 1D and 1E). As an introspective and dialogic process that can support transformative education, conscientization has great relevance for the decolonization of GN [33].

## Discussion

The preceding vignettes highlight the impact of colonialism on people of African descent worldwide, including the contemporary GN inequities that have emerged as a component of broad global surgery- and other health system-related inequities. Beyond revealing thematic convergence of parallel histories, they sketch a global narrative of coloniality that is linked to current neurosurgical inequities in these countries, and many others not described here. This shared narrative of health inequity imbues GH and its subspecialties, such is GN, with transformative potential that may be realized through education that prioritizes: (1) understanding the depth, breadth, and heterogeneity of colonial history, (2) recognising and studying the colonial legacy as a determinant of contemporary forms of structural violence, structural racism and inequity in global health and its subspecialties such as GN, and (3) proactively working to decolonize GN itself, understanding that without proactive decolonization, the field risks failing to achieve its stated primary purpose: to ensure access to essential neurosurgical care to *all* who need it [96].

### The challenge of decolonization

Coloniality in GH is all-pervasive and its solutions remain elusive for several reasons. First, the colonial experiences in each postcolonial society are distinct; effective capacity-building work in GN therefore requires careful reflection on the individual colonial histories of target populations and their distinctive impact on neurosurgical capacity in each postcolonial LMIC [96]. For instance, the linguistic division between English-speaking and French-speaking Cameroon can have significant implications for capacity-building efforts; ignorance about this cultural particularity and its colonial roots can interfere with the effectiveness of well-intended GN initiatives. Secondly, discerning coloniality in contemporary GN is particularly challenging when HIC stakeholders are not the actors who generated colonial structures, but rather their descendants. This intergenerational gap can hinder the ability of GN practitioners from dominant social groups to fully recognize and empathize with the realities of the people they seek to serve [96]. Thirdly, coloniality influences every aspect of social life in postcolonial societies, including education; even the most educated members of any society may therefore suffer from a an epistemological bias that prioritizes education from the dominant or power-wielding group. Consequently, GN actors from HICs will often have received formal historical and other social science education that neglects critical perspectives from LMIC populations. The inadequacy of this training for effective GN work underscores the importance of developing broad GH competencies as part of a culturally humble and decolonial approach to GN practice [97].

Training in disciplines comprising foundational GH education in GN, such as history, medical anthropology, and the socio-behavioural sciences of public health, can inspire the cultural humility required for decoloniality [98]. For instance, when the morbimortality of neurosurgical inequities is recognized as a form of structural violence rooted in the colonial legacy, neurosurgeons are empowered to generate solutions that address the unjust structures perpetuating these inequities. Treatment of a particular disease in a target LMIC, like hydrocephalus, can certainly be partially addressed through humanitarian efforts ranging from mission-style volunteering initiatives to education-focused projects. However, if morbimortality from hydrocephalus stems from structural problems such as poverty, socioeconomic inequality, political instability, and lack of access to education or basic health services, then the persistence of these structures will limit the success of GN endeavours [99].

### The way forward

#### Conscientization and anti-racism as decolonial praxes

Modern neurosurgical inequities are a form of health system-related structural violence in that they discriminate by generating both the privileges of the most fortunate, who enjoy access to neurosurgical care, and the misfortune of the destitute sick who suffer from lack of such access. Recognizing GN inequities as a form of structural violence may empower GN stakeholders to channel humanitarian motivations into a decolonial humanism. Conscientization and anti-racism are complementary approaches that can support reflective, transformative practices that inspire this decolonial humanism [32–35].

#### Dialogic conscientization

Conscientization may be implemented by GN practitioners through deliberate reflection primed by acknowledging the existence and influence of colonial power dynamics in GN relationships [33]. In the process of locating themselves within sociohistorical hierarchies, GN stakeholders can initiate transformative dialogues that enable decoloniality. For instance, when HIC neurosurgeons meet their LMIC counterparts, the former may grow in cultural humility from conversations that enable them to see how the randomness of place of birth thrusts HIC partners into the (neo-)-colonial role of “oppressor” vs. the LMIC partner’s role as, “oppressed.” [35]

When the racially, socially and/or economically disadvantaged position of “LMIC partner” results in that partner’s absence from conversations that shape her/his/their healthcare reality, default approaches to problem-solving structurally perpetuate the colonial legacy. Enabling historically disempowered voices to shape our GH dialogues can instantly transform traditional power dynamics, and advance equitable, decolonial solutions to GH problems by liberating HIC and LMIC partners from the inherited, dichotomous colonial roles of “oppressor” and “oppressed” [35].

Moving beyond this praxis and its inherent shifting frames of reference, GN actors may further engage in conscientization through introspection exploring the colonially inherited pretence that the privilege of whiteness, or the disadvantage of blackness, confers real gradations of superiority or inferiority onto human beings [33]. This level of introspection can further inform authentic cross-cultural communication that nurtures decolonial GN partnerships, particularly when a common language, centring the native tongue of the LMIC partner, is prioritized to enable an equitable dialogic plane.

#### Anti-racism

Anti-racism may be thought of as a corollary to the transformative process of conscientization, and is any form of opposition to racism or a racist structure [32, 34]. The concept provides an actionable posture for dismantling structural inequities that underlie GN disparities, and it may take the form of proactive advocacy for policies and practices that protect and empower people from disenfranchised racial groups, such as countering surgical colonialism in clinical endeavours abroad, or deferring first- and senior-authorship roles in GN manuscripts to LMIC partners and racially marginalized actors [3, 97]. Anti-racism may also take the form of advocacy against racist laws or policies that discriminately harm people from underserved groups, which in many societies are “black” people of presumed African ancestry [32]. For instance, neurosurgeons in the USA who care for victims of penetrating brain trauma can be influential anti-racist advocates for gun control in the face of data revealing that black men in that country are 14 times more likely to be killed by firearm than white men [100].

Table 4 presents a guide for reflection, dialogue and project assessment in GN endeavours. Based upon the introspective and dialogic practices recommended above, we are hopeful that it will serve as a practical tool that facilitates decolonization in modern GN.

**Table 4:** A guide for reflection, dialogue and project analysis to decolonize global neurosurgery.

**In scientific endeavours:**
• Will authorship order and work distribution empower the team members who represent the marginalized population? • Is the research question primarily shaped by local priorities? • What impact will publications have on the local culture and society versus the careers and home institutions of HIC stakeholders? • Does the publication plan accommodate the local language and accessibility requirements that maximize the usefulness of the publication to LMIC partners and stakeholders? • Are there disparities in research capacity that would be addressed with initiatives that prioritize the advancement of scientific equity? • Do local IRBs exist? Have local IRBs reviewed the ethical soundness of the research plan?
**In clinical educational initiatives:**
• What neo-colonial dynamics are introduced into new international educational relationships and partnerships? • Have local leaders in existing clinical education initiatives been identified and engaged in educational processes? • Have local needs and preferences been comprehensively solicited, fully understood, and authentically prioritized in the development of educational programs? • What is/are the language(s) of instruction and how are they being used? • How are institutional partnerships being generated, and what pathways for bidirectional education have been explored? • Examine underlying assumptions of the power dynamics in international education partnerships (teacher/student, attending/resident, mentor/mentee): Are HIC practitioners or experts explicitly, or implicitly assumed to be “superior” to LMIC practitioners or experts? • What steps can be taken to dismantle neo-colonial power structures of the project or initiative?

A practical guide for GN partnerships to support reflection, dialogue and project analysis that prioritizes decolonial humanism in GN and GH.

## Conclusion

GN is an evolving interdisciplinary subspecialty that entails clinical and public health practice aimed at achieving GH equity for all people worldwide who require essential neurosurgical care. As a field that has emerged and been driven by neurosurgeons in HICs seeking to serve the needs of people in LMICs, the goals of the subspecialty are threatened by the spectre of a colonial legacy characterized by the exploitation and dehumanization of people in Sub-Saharan Africa and the African Diaspora. The resulting institutions can only be successfully recognized and transformed through a deliberate process of education about colonial history and its neo-colonial manifestations, a new dialogue that restores power and humanity to the very people who have suffered from resulting structural racism and structural violence, and GN initiatives that are developed in an authentically inclusive, anti-racist and culturally humble manner [31].
